# The Utility of Physiological Measures in Assessing the Empathic Skills of Incarcerated Violent Offenders

**DOI:** 10.1177/0306624X21994056

**Published:** 2021-02-11

**Authors:** Julie Palix, Ahmad Abu-Akel, Valérie Moulin, Milena Abbiati, Jacques Gasser, Christopher Hasler, Dominique Marcot, Christine Mohr, Elise Dan-Glauser

**Affiliations:** 1 Centre Hospitalier Universitaire Vaudois, Lausanne, Switzerland; 2 Institute of Psychology, University of Lausanne, Switzerland

**Keywords:** violence, offenders, empathy, heart rate variability, RMSSD

## Abstract

Since lack of empathy is an important indicator of violent behaviors, researchers need consistent and valid measures. This study evaluated the practical significance of a potential physiological correlate of empathy compared to a traditional self-report questionnaire in 18 male violent offenders and 21 general population controls. Empathy skills were assessed with the *Interpersonal Reactivity Index* (IRI) questionnaire. Heart-Rate Variability (HRV) was assessed with an electrocardiogram. The RMSSD (Root Mean Square of the Successive beat-to-beat Differences), an HRV index implicated in social cognition, was calculated. There were no group differences in IRI scores. However, RMSSD was lower in the offender group. Positive correlations between RMSSD and IRI subscales were found for controls only. We conclude that psychometric measures of empathy do not discriminate incarcerated violent offenders, and that the incorporation of psychophysiological measures, such as HRV, could be an avenue for forensic research on empathy to establish translatable evidence-based information.

## Introduction

In court decisions, offenders’ empathic skills are often taken into consideration to determine offenders’ incarceration length, release from prison, and risk of recidivism (Campbell & Schmidt, 2000). They are also considered when evaluating rehabilitation progress in correctional settings (Jolliffe & Farrington, 2004; Marlow et al., 2012). In the context of the justice system, empathic skills are typically conceptualized in terms of whether the offender feels remorse, has empathic concern for the victim, and is cognizant of the effect of crime on society. Typically, assessments of offenders’ empathic skills have largely relied on self-report instruments (Jolliffe & Farrington, 2004; Robinson & Rogers, 2015; van Langen et al., 2014). The aim of our study is to assess the practical significance of a potential physiological correlate of empathy in comparison to a traditional self-report questionnaire in incarcerated male violent offenders.

Empathy is generally defined as the ability to recognize other people’s thoughts and feelings and to respond to these with an appropriate emotion (Shamay-Tsoory, 2011). Models of empathy (Davis, 1980; Gladstein, 1983; Kerem et al., 2001; Lawrence et al., 2004) generally agree that empathy consists of i) a cognitive component, that is, the ability to consider another’s viewpoint, and ii) an affective component, that is, the ability to vicariously share another person’s emotional experience. Empathy is considered central to functional interpersonal relationships (Eisenberg et al., 2002; Miller & Eisenberg, 1988) and prosocial behavior (Eisenberg & Fabes, 1990). Empathy has also been viewed as an inhibitor of aggressive and violent behavior (Feshbach & Feshbach, 1982; Jolliffe & Farrington, 2006; Miller & Eisenberg, 1988; Richardson et al., 1994, 1998), and it was suggested that aggressive and violent behavior may result from a deficit in empathy (Abu-Akel & Bo, 2018; Blair, 2005). In this regard, Jolliffe and Farrington (2006) noted that “during a violent interaction, the emotions of the victim are clearly available to the perpetrator and an inability to react to these emotions is evidence of a lack of empathy” (p. 546). Thus, it can be hypothesized that, compared to non-offenders, offenders, and particularly incarcerated violent offenders, may present lower levels of empathy.

Research on the relationship between empathy and aggression in offenders has largely been based on male forensic populations (Jolliffe & Farrington, 2004; van Langen et al., 2014), who typically exhibit elevated levels of psychopathic traits, characterized by a general lack of empathy and remorse (Coid et al., 2009). Research into the relationship between low empathy and high aggression in offenders has, however, yielded heterogeneous results (Baly & Butler, 2017; Beven et al., 2004; Vachon et al., 2014). Based on self-report questionnaires, some studies found that offenders have lower empathy levels than controls (Domes et al., 2013; Jolliffe & Farrington, 2004; Mariano et al., 2017). For example, Domes et al. (2013) found that relative to male controls, male offenders exhibited lower cognitive and affective empathy. In contrast, other studies found no differences in general empathy scores (Farr et al., 2004; McGrath et al., 1998; Monto et al., 1994). For example, Farr et al. (2004) found no difference in the empathic abilities of male adolescent offenders as compared to male adolescent non-offenders.

Three main hypotheses have been proposed to explain this heterogeneity. First, different components of empathy might differentially discriminate violent offenders and non-offenders (Abu-Akel & Abushua’leh, 2004; Gantiva et al., 2018; Jiang et al., 2019; van Langen et al., 2014). For example, a meta-analysis found that cognitive empathy was more strongly associated with offending than affective empathy (van Langen et al., 2014). Second, the majority of studies has relied on self-report questionnaires to measure empathy, which might be problematic (Irvine & Gendreau, 1974; Jolliffe & Farrington, 2004; Robinson & Rogers, 2015; West, 1988). For example, empathy self-report questionnaires have been shown to lack accuracy and to be prone to socially desirable responding (Curwen, 2003; Domes et al., 2013; Kampfe et al., 2009; Tierney & McCabe, 2001), and that their generalizability to populations in clinical or legal settings seems limited (Hanel & Vione, 2016). Third, some offenders may be adept at masking or camouflaging their empathic deficits. Studies have shown that participants can learn what empathy is and be able to respond in an acceptable fashion (Day et al., 2010; Koegel et al., 2016; Muñoz et al., 2012), and that offenders are capable to simulate high levels of empathy quite easily (Robinson & Rogers, 2015).

Given inherent limitations in administering self-report empathy questionnaires to offender populations, it would be important to establish measures of empathy that are ideally not under the participants’ control (Gerdes et al., 2010; Kampfe et al., 2009; Mohr et al., 2013; Nosek et al., 2011; Vachon et al., 2014). This would improve the reliability and validity of the measurement of empathy skills (Jolliffe & Farrington, 2004; Robinson & Rogers, 2015), and would therefore be of enhanced significance for judicial and forensic purposes (Curwen, 2003; Young et al., 2008).

Neuroscientific studies have uncovered mechanisms that may underlie human empathic abilities (for review see Decety & Svetlova, 2012), and suggest that the social brain develops at an early age, meeting the need for parental care and bonding with group members to facilitate survival. The neural mechanisms involved in these social dynamics serve intrinsically important psychophysiological regulatory functions, and include the stress-related hypothalamic-pituitary-adrenal axis (HPA axis), the fight-or-flight sympathetic (SNS), and the well-being parasympathetic (PNS) components of the autonomic nervous system (Carter, 1998; Carter & Porges, 2011). The SNS involves fear, anxiety and reactive behaviors to stressful stimuli, and the PNS promotes rest, mind-reading, attachment and prosocial engagement (Porges, 2001, 2007). The balance between these two antagonist systems is achieved in childhood, reorganized in adolescence, and reaches maturity in early adulthood (Alink et al., 2008; Platje et al., 2013). One promising psychophysiological measure for the evaluation of this sympathetic-parasympathetic balance is the vagally mediated variation of the inter-beat intervals observed in the continuous heart rate recordings, also known as heart rate variability (HRV) (Appelhans & Luecken, 2006; Berntson et al., 1991). Using indices of HRV, robust associations have been made between variability in very low-frequency—indicative of the SNS excitatory activity—and aggressive, hostile, and anti-social behaviors (Beauchaine et al., 2007; Demaree & Everhart, 2004; Mezzacappa et al., 1997; Palix et al., 2015, 2017; Sloan et al., 2001), as well as between variability in high-frequency—indicative of the PNS inhibitory activity—and social skills (e.g., number of supportive friends, empathic responses to the suffering of others) (Geisler et al., 2013; Holt-Lunstad et al., 2007; Lischke et al., 2018; Miller et al., 2020; Quintana et al., 2012; Schwerdtfeger & Schlagert, 2011). Indeed, it has been reported that individuals with high PNS are more likely to understand and share mental and emotional states, and to be empathetic, than those characterized by low PNS activity (Kok & Fredrickson, 2010; Lischke et al., 2018). These measures are obtained at the resting state, during which the autonomic system is at its baseline level, without contextual or individual interference in response to a given situation (e.g., avoidance) (Miller, 2018).

The aim of our study is, therefore, to assess the practical significance of HRV as a physiological assessment of empathy in comparison to a traditional self-report empathy questionnaire, in a population of male violent offenders. To this end, we contrasted both measures in incarcerated male violent offenders and in a male control group from the general population. In line with previous research, we expected that offenders would score lower on the self-report empathy questionnaire, and that inter-individual differences in PNS activity at rest would be positively associated with inter-individual differences in self-reported empathy (Lischke et al., 2017; Lischke et al., 2018; Miller & Eisenberg, 1988). Second, we investigated how the associations between self-reported empathy and HRV indices differed between the two groups. We predicted that both self-reported empathy and variability in the high-frequency index of HRV (indicative of PNS activity) would be *lower* in the offender group, whereas the variability in the very low-frequency index of HRV (indicative of SNS activity) would be *higher* in the offender group.

## Methods

### Participants

Participants were all male and consisted of an offender and a control group. The final sample in the offender group consisted of 18 males, mean age = 39.5 (always in years), standard deviation (*SD*) = 13.2 years, range 24 to 72. Originally, we had contacted 46 offenders. Forty-four were prisoners at a local high-security prison. A total of 20 registered for our testing sessions, 10 refused to participate, 6 had an insufficient command of French, 2 could not be tested (security situation such as solitary confinement), 2 had left the institution, and 2 had denied the offense. Two additional offenders volunteered after recruitment via an outpatient consultation (Centre Hospitalier Universitaire Vaudois, CHUV, Lausanne). Of these 22, four further participants had to be excluded because of technical difficulties in the measurements (see the Data processing and statistics section). We offered no financial compensation to avoid unequal treatment due to our inclusion and exclusion criteria. We included participants who were at least 18 years of age, had committed one or more violent offences against another person, and were French speakers. We excluded participants who had committed other types of offences (e.g., property or sexual offences), awaited trial, or who could not be tested with an electrocardiogram (e.g., due to health problems). Other characteristics of the offender group are described in Table 1.

**Table 1.** Characteristics Specific to the Offender Group.

Measures	Label	*N*	%
Diagnosis	None	6	33
	Personality Disorder	8	45
	Psychosis	4	22
Psychiatric follow-up	Yes	4	22
	No	14	78
Victim known to offender	Yes	13	72
	No	5	28
Principal offence	Homicide	11	61
	Homicide attempt	5	28
	Other forms of violence	2	11
Type of violent act^a^	Impulsive	13	72
	Premeditated	5	28
Measures	*M*	*SD*	[minimum; maximum]
Sentence duration (years)	10.2	5.3	[1; 20]
Number of infractions	2.06	1.2	[1; 5]
Time between crime and testing (years)	8.6	8.0	[3; 32]

a Based on information from the psycho-criminal interview.

The control group consisted of 21 males, mean age = 30.2, *SD* = 10.9, range 19 to 57. Control participants were recruited to correspond to the offenders in terms of age and socio-economic status. All were tested with the same protocol as the offender group, in a half-light, quiet room, alone with the experimenter. The test either took place in a room in the university hospital, or in one of the army trainee garrisons. In a first step, six young men were recruited on the Swiss Army compulsory enlistment days in Lausanne. This recruitment lasts 3 days, and it is obligatory for all young Swiss men. Therefore, they are representative of the Swiss male population, and come with diverse levels of physical fitness. For the duration of the recruitment, they must stay on site and sleep in a shared dormitory. The recording session of the experiment was proposed on the first or the second day. Participation was voluntary. Due to their young age (mean age of 19), the group was supplemented by older volunteers through announcements targeted to people between 30 and 60 years of age, of average socio-economic status, in good health, and with no criminal record for a violent offence against another person. These criteria were confirmed in a personal interview. Of the 24 participants originally recruited, 3 were excluded because of technical difficulties in recording their data (see the Data processing and statistics section), leaving 21 control participants in the sample. Participants were reimbursed for their expenses (40 Swiss Francs). The army participants were not compensated on request of the administration. Table 2 shows the distribution of education level, nationality, age, and body mass index (BMI) in the offender and control groups.

**Table 2.** Demographic Data of the Control (C) and Offender (O) Groups.

Measures	Group	Characteristic	*N*	% in group
Education level	C / O	No education	2 / 3	10% / 17%
		Elementary education	11 / 10	52% / 55%
		Higher education	8 / 5	38% / 28%
Nationality	C / O	Swiss	16 / 11	75% / 61%
		European	5 / 6	25% / 33%
		Other	0 / 1	0% / 6%
Measures	Group	Mean	*SD*	*t*–test
Age	C	30.2	10.9	***t*_(37)_** = 2.41*, *d* = **0.78**
	O	39.5	13.3	
Body mass index	C	23.0	1.87	*t*_(37)_ = −1.73, *d* = −0.56
	O	25.1	5.40	

* *p* = .02.

### Measures

#### Characteristics of the criminal offense and sentence

We assessed each offender’s criminal and psychopathological profile by examining criminal records and through semi-directive interviews. From the criminal records, we recorded the number of crimes for which the offender was arrested, the main crime committed, whether the victim was known to the offender, the length of the sentence, and whether a psychiatric follow-up was ordered by the court. During the semi-directive interview, we assessed the contextual circumstances of the offence and the way the crimes were perpetrated, and specifically the triggering event, and the premeditated or impulsive nature of the assault (Moulin & Senon, 2010). The psychiatric diagnosis was based on the one given by the psychiatric referents during the penal expertise or during incarceration.

##### Interpersonal Reactivity Index (IRI) questionnaire

This 28-items questionnaire consists of four subscales of seven items each (Davis, 1980, 1983). Since each of the subscales evaluates an independent component of empathy (Davis, 1983; D’Orazio, 2004; Eisenberg & Fabes, 1990), they were evaluated separately. The first subscale is Perspective Taking, assessing the ability to adopt others’ point of view. An example item is “Before criticizing somebody, I try to imagine how I would feel if I were them.” The Fantasy subscale assesses the ability to experience the emotional states of fictional characters. An example item is “I really get involved with the feelings of the characters in a novel.” The Perspective Taking and the Fantasy subscales tap into the cognitive dimension of empathy. The Empathic Concern subscale assesses the feeling of sympathy toward others. An example item is “I often have tender, concerned feelings for people less fortunate than me.” Finally, the Personal Distress subscale assesses participants’ avoidance of negative feelings or discomfort when interacting with a person feeling distressed. An example item is “Being in a tense emotional situation scares me.” The Empathic Concern and the Personal Distress subscales tap into the emotional dimension of empathy. Participants indicate to what extent they agree with the respective statement on a four-point Likert scale ranging from 0 to 4. Nine items are reverse coded. The scores are then summed for each subscale, and range from 0 to 28. Higher scores indicate a higher level of empathy. We used the validated French version (Gilet et al., 2013). This IRI scale has been extensively used in the general population (Davis, 1980), as well as in the offender population (Daffern et al., 2018; Mayer et al., 2018; Polaschek & Reynolds, 2004), and shows good and reliable psychometric properties, across cultures and languages (Albiero et al., 2006; Gilet et al., 2013).

##### Heart rate variability (HRV)

Resting state Heart rate variability (HRV) was recorded independently for a period of 5 minutes, prior the experimental tasks. Recording of HRV in a resting state makes it easier to avoid contamination by the autonomic reaction that occurs during an emotional situation (Deuter et al., 2018). The heart rate of each participant was recorded using a commercially available wireless electrocardiogram (ECG, Equivital system, Cambridge, UK and Vivosense^®^ by Vivonoetics Inc, Newport Coast, USA). This ECG system consisted of a chest-belt containing sensors to be placed on the skin, and a Sensor Electronics Module (SEM) box connected via Bluetooth to a laptop that recorded the data at an acquisition rate of 250 Hz. The resting state data were then exported offline in an ASCII format and processed with Kubios HRV Premium software version 3.0.2 (Kubios Oy, 2016–2019, https://www.kubios.com, Kuopio, Finland; Tarvainen et al., 2014). Processing included de-trending, removal of artifacts, and the extraction of HRV parameters (Lipponen & Tarvainen, 2019; Tarvainen et al., 2002).

The HRV parameter representative of the inhibitory PNS activity is the time-domain Root Mean Square of the Successive Differences (RMSSD), in milliseconds (Camm et al., 1996; Zygmunt & Stanczyk, 2010). The higher this index is, the stronger the parasympathetic activity is considered to be. The short-term norm of RMSSD in the general population averages 42 ms ± 15 (Nunan et al., 2010). We also measured the excitatory SNS activity using the absolute proportion of the Very Low Frequency band (VLF: 0.0033–0.04 Hz, Fast Fourier Transform) from the total spectral power (McCraty & Shaffer, 2015). Because the sympathetic activity is considered slow, on the range of seconds, its time-domain analysis is not recommended. This frequency-domain estimate is provided in the results tab generated by Kubios software (Kubios Oy, 2016–2019, https://www.kubios.com, Kuopio, Finland).

### Procedure

The experimenter selected potential participants for the study on the basis of prison records and inclusion criteria. Then, participants received a written description of the study and were given time to think about their potential participation. Participants provided a signed consent form before the study began. For offenders undergoing a psychiatric follow-up, we informed their medical referees about the study. The medical referees did not play any further role in the study. Participants were invited to take part in two separate sessions, on different days.

In the first session, participants were first invited to ask any question about the study. Then, the belt with the ECG sensors was comfortably positioned in a closed, shaded room. The prison security service remained outside the room in order to secure the participants’ separation from their detention conditions. The participants then performed two computerized tasks: (i) an emotion detection threshold task (Rodger et al., 2015) and (ii) a Go-No-Go task (Gomez et al., 2007; Gordon & Caramazza, 1982) during which their ECG was recorded, for a total duration of about 40 minutes. The ECG recording also included two 5-minute rest-periods, eyes-opened, before and after performing the experimental tasks. Experimental results of the two tasks are reported elsewhere and are not considered in the present publication. The belt was then removed, the light was turned on, and the participants filled the IRI questionnaire in the presence of the experimenter. In addition, the participant filled the Impulsive Behavior Scale (Whiteside & Lynam, 2001) and the Emotion Reactivity Scale (Nock et al., 2008), both for another study. The whole session lasted about an hour including the installation and removal of the belt.

In the second session, we performed the psycho-criminological assessment in the form of a clinical interview, semi-directive, structured in its course, and chronologically constructed around the offence (before, during, and after) (Moulin & Senon, 2010). This interview was conducted by an advanced clinician, one on one with the participant, in a guarded office. This interview lasted for an approximately 1 hour.

This study was carried out in accordance with the World Medical Association declarations of Helsinki, and was approved by the Ethics Committee of the Faculty of Biology and Medicine of the University of Lausanne (CER-VD 58/14).

### Data Processing and Statistics

For the calculation of HRV parameters in the control group, two datasets were unusable due to technical problems with the recording. Automatic correction of artifacts—proposed by Kubios Premium—was applied to the data of 16 participants, medium correction to the data of 2 participants, and strong filtering to the data of 4 participants. In the offenders group, one dataset was unusable due to incomplete recordings. Automatic correction was applied to the data of 16 offender participants, medium correction to the data of 3 participants, and strong filtering to the data of 2 participants. In one of these two cases, the remaining data were considered to be insufficient for further analyses and was therefore excluded. In means, 4 beats ± 7 (1.25%) were corrected in the control group for an average of 3 minutes ± 1*SD* of data length, and 3 beats ± 3 (1.67%) were corrected in the offender group for an average of 2 minutes 50 seconds ± 1*SD* of data length. For seven participants (four in the offender group) we had either no valid physiological recordings or no IRI scores. All analyses were thus performed on 18 offenders and 21 control participants (see the Participants section above).

We used independent t-tests to compare the groups on age, body mass index, IRI sub-scale scores, and heart rate variability measures. Chi-square tests were used to compare the frequencies of education levels and nationality across the two groups. We also calculated Pearson’s correlations to evaluate the relationship between IRI and HRV parameters for the full sample and each group, separately. IRI subscales showing significant correlations (see Table 4) with HRV parameters were then further investigated in multiple linear hierarchical regression models. Specifically, in two separate analyses, we investigated the association of Perspective Taking and Empathic Concern with RMSSD, while controlling for age, BMI, and the group factor (Offenders vs. Controls). We controlled for age and BMI since the two groups differed in age (see Table 2) and because BMI correlated with RMSSD (see correlation analyses in the results section). For each of these models, we entered in Step 1 age and BMI, in Step 2, the group factor; in Step 3, the scores of the corresponding IRI subscale; and in Step 4, the interaction between the group factor and the scores of the IRI subscale. For each model, we report the *F* change statistics, which reflects the additional amount of variance explained due to the inclusion of variables in subsequent steps. In case of a significant interaction between the group factor and the IRI subscale, we performed two follow-up simple regressions examining the association of the IRI subscale scores with RMSSD, separately in the control and the offender groups. Finally, we used Mann-Whitney *U* Tests to explore potential clinical and demographic difference between offender subgroups based on their response pattern on the IRI. Effect sizes are reported in terms of Cramer’s *V*, Cohen’s *d*, and *R*^2^, as appropriate.

## Results

### Socio-Demographic Information

The offender group was, on average, older (*t*_(37)_ = 2.41; *p* = .02, *d* = 0.78). There were no differences between the groups in the distribution of nationality (χ^2^_(2)_ = 1.80, *p* = .41, *V* = .22) or education level (χ^2^_(2)_ = 0.71, *p* = .70, *V* = .14) (see details in Table 2).

### IRI Scores and HRV between Groups

Table 3 summarizes the differences between the offender and the control groups in IRI scores and HRV values. The two groups had comparable empathy self-report scores. For HRV measures, the offender group had a significantly lower RMSSD (the parasympathetic index) (*p* = .007, Cohen’s *d* = 0.93), and a significantly higher percentage of VLF (the sympathetic index) (*p* < .001, Cohen’s *d* = −1.56).

**Table 3.** Mean, Standard Deviation (*SD*) and 95% Confidence Interval (95% CI) for the Self-Report Empathy Subscales and Heart Rate Variability Measures for Each of the Study Groups.

Measures	Group	Mean	*SD*	95% CI	*t*-test, *p*-value	Cohen’s *d*
IRI perspective taking	Controls	17.08	6.08	[14.65; 19.51]	*t*_(42)_ = 0.46, *p* = .65	0.14
	Offenders	16.30	4.91	[14.15; 18.45]		
IRI fantasy	Controls	13.92	5.43	[11.74; 16.09]	*t*_(42)_ = 0.62, *p* = .54	0.19
	Offenders	12.95	4.74	[10.87; 15.03]		
IRI empathic concern	Controls	17.92	5.22	[15.83; 20.00]	*t*_(42)_ = −0.67, *p* = .50	−0.21
	Offenders	19.05	5.94	[16.45; 21.65]		
IRI personal distress	Controls	8.29	4.10	[6.65; 9.93]	*t*_(42)_ = −0.63, *p* = .53	−0.19
	Offenders	9.20	5.48	[6.80; 11.60]		
Heart rate	Controls	73.93	10.97	[68.94; 78.92]	*t*_(37)_ = −1.04, *p* = .305	−0.34
	Offenders	78.17	14.43	[70.99; 85.34]		
RMSSD (msec)	Controls	41.79	21.41	[32.64; 50.95]	***t*_(38)_** **=** **2.87, *p*** **=** **.007**	**0.93**
	Offenders	25.52	12.95	[19.70; 31.34]		
Very low frequency (absolute power proportion)	Controls	23.89	17.70	[16.32; 31.45]	***t*_(37)_** **=** **4.74, *p*** **<** **.001**	**−1.56**
	Offenders	51.28	18.89	[43.48; 61.81]		

*Note.* The results of the group comparisons and Cohen’s d for each test are given (significant *p*-values in bold). IRI = Interpersonal Reactivity Index; RMSSD = Root mean square of successive heart interbeat differences.

### Correlations between IRI Subscales, HRV Measures, Age, and BMI

**Figure 1.**
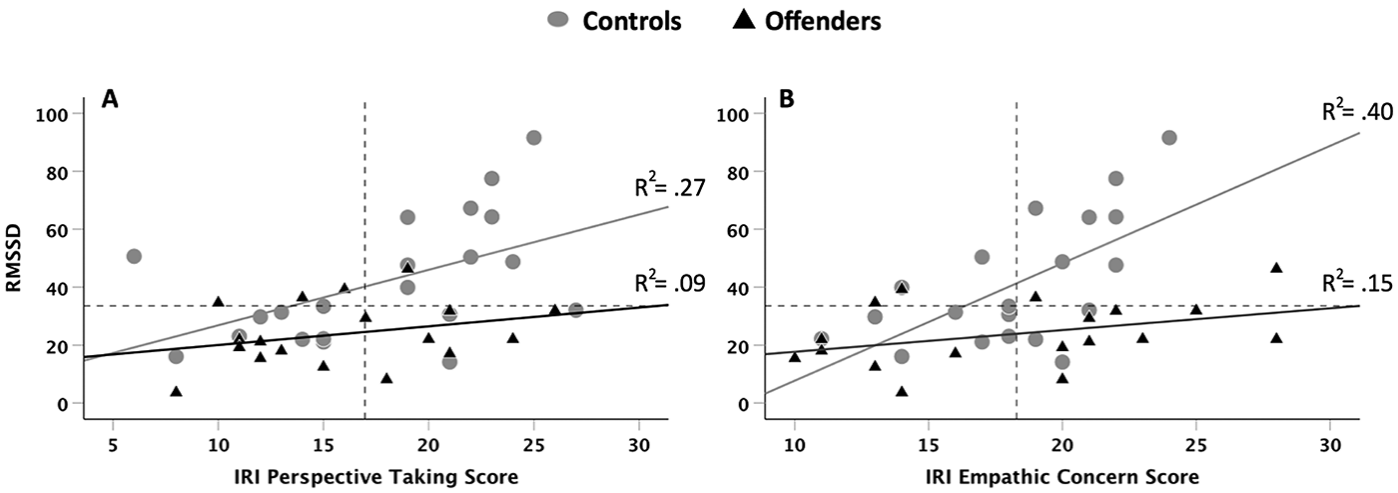
The relationship between self-reported empathy (Interpersonal Reactivity Index, IRI) and the parasympathetic index (root mean square of successive heart interbeat differences, RMSSD) in control (gray dots) and offender (black triangles) groups. Panel A depicts the relationship between Perspective Taking scores and RMSSD (ms), and panel B depicts the relationship between Empathic Concern scores and RMSSD (ms). The relationship of RMSSD with Perspective Taking (*r* = .52; *p* = .017) and with Empathic Concern (*r* = .63; *p* = .003) was positive and only significant in the control group. In both panels, dashed horizontal lines represent the overall mean for RMSSD (Mean = 33.55 ms), and dashed vertical lines represent the mean scores of Perspective Taking (Mean = 16.97) and Empathic Concern (Mean = 18.29).

In the overall sample, Pearson’s correlations (see Table 4) showed that RMSSD values significantly and positively correlated with both Perspective Taking (*r* = .46, *p* = .003) and Empathic Concern (*r* = .40, *p* = .013). Interestingly, for both subscales (see Figure 1), these correlations were significant in the control group (Perspective Taking, *r* = .52, *p* = .017; Empathic Concern, *r* = .63, *p* = .003), but not in the offender group (Perspective Taking, *r* = .29, *p* = .239; Empathic Concern, *r* = .38, *p* = .115). The remaining correlations were not significant. Since RMSSD was significantly correlated with VLF (*r* = −.367, *p* = .022), we examined, in partial correlations, the association of RMSSD with the IRI subscales, while controlling for variation in VLF values. We obtained similar results. In the overall sample, RMSSD significantly and positively correlated with both Perspective Taking (*r* = .53, *p* < .001) and Empathic Concern (*r* = .40, *p* = .015), and as before, these associations were significant only in the control group (Perspective Taking, *r* = .64, *p* = .003; Empathic Concern, *r* = .63, *p* = .004). No other significant associations were observed. Consequently, we did not further consider the very low frequency measure and the Fantasy and Personal distress subscale scores in the regression models.

**Table 4.** Pearson’s *r* for the Relationships Between Self-Reported Empathy and Measures of Heart Rate Variability.

Groups	All (*N* = 39)		Offenders (*N* = 18)		Controls (*N* = 21)	
Measures	RMSSD	VLF	RMSSD	VLF	RMSSD	VLF
Self-reported measures						
IRI perspective taking	.46**	−.23	.29	−.33	.52*	−.02
IRI fantasy	.13	−.07	−.44	.20	.25	−.10
IRI empathic concern	.40*	−.20	.38	−.27	.63**	−.24
IRI personal distress	−.10	−.14	−.19	−.27	−.06	−.14

*Note.* IRI = Interpersonal Reactivity Index; RMSSD = Root mean square of successive heart interbeat differences; VLF = Very Low Frequency.

Bold values are significant at *p* < .05.

* *p* < .05. **p < .01.

Age did not significantly correlate with any of the IRI subscales or HRV measures (*p*s > .05). However, there was a significant negative association between BMI and Perspective Taking in both the overall sample (*r* = −.42, *p* < .01) and the offender group (*r* = −.54, *p* < .01), and with RMSSD in the control group (*r* = −.44, *p* < .05).

### Hierarchical Regression Analyses of Empathy on Parasympathetic HRV Index RMSSD

#### Perspective taking scores

The results of the hierarchical model are summarized in Table 5. In Step 1, neither age nor BMI were significant predictors. Step 2 revealed a significant group main effect, with the offender group showing lower RMSSD values than the control group. In Step 3, entering the Perspective Taking scores revealed a significant positive association with the RMSSD, and improved the model by explaining an additional 14% of the variance. In Step 4, however, entering the interaction of Group x Perspective Taking scores did not significantly improve the model, *F*_change_(1, 33) = 1.44, *p* = *.239, R*^2^_change_ = .03 (see also Figure 1a). The model, in Step 3, explained a total of 39% of the variance in RMSSD.

**Table 5.** Hierarchical Regression Analyses on RMSSD With the Self-Reported Perspective Taking Subscale.

Predictors	*B*	*SE*	β	*p*	*F*(*df*)	*p* of *F*	*R* ^2^	Δ*F* (*df*)	Δ*R*^2^	*p* of Δ*F*
Step 1					2.91 (2,36)	.067	.14			
Age	−0.45	0.24	−0.30	.064						
BMI	−0.99	0.75	−0.20	.197						
Step 2					3.89 (3,35)	.017	.25	5.16 (1,35)	.11	**.029**
Age	−0.25	0.24	−0.17	.296						
BMI	−0.55	0.74	−0.11	.463						
Group	−14.30	6.29	−0.37	**.029**						
Step 3					5.41 (4,34)	.002	.39	7.74 (1,34)	.14	**.009**
Age	−0.23	0.22	−0.15	.304						
BMI	0.26	0.74	0.05	.729						
Group	−13.63	5.77	−0.35	**.024**						
Perspective taking	1.47	0.53	0.41	**.009**						
Step 4					4.67 (5,33)	.002	.42	1.44 (1,33)	.03	.239
Age	−0.22	0.22	−0.15	.317						
BMI	−0.08	0.78	−0.02	.919						
Group	8.01	18.92	0.21	.675						
Perspective taking	1.87	0.62	0.53	**.005**						
Group × perspective taking	−1.26	1.05	−.57	.239						

*Note.* RMSSD = Root mean square of successive heart interbeat differences; BMI = Body Mass Index; Group = Controls or Offenders.

Bold values are significant at *p* < .05.

#### Empathic concern scores

The results of the hierarchical model are summarized in Table 6. In Step 1, neither age nor BMI were significant predictors. Step 2 revealed a significant group main effect of the group, with the offender group showing lower RMSSD values than the control group. In Step 3, entering the Empathic Concern scores revealed a significant positive association with the RMSSD, and improved the model by explaining an additional 16% of the variance. In Step 4, entering the interaction of Group x Empathic Concern scores, significantly improved the model by an additional 13%. This final model explained a total of 52% of the variance in RMSSD (see also Figure 1b).

**Table 6.** Hierarchical Regression Analyses on RMSSD With the Self-Reported Empathic Concern Subscale.

Predictors	*B*	*SE*	β	*p*	*F*(*df*)	*p* of *F*	*R* ^2^	Δ*F* (*df*)	Δ*R*^2^	*p* of Δ*F*
Step 1					2.52 (2,35)	.095	.13			
Age	−0.43	0.24	−0.28	.086						
BMI	−0.97	0.76	−0.20	.214						
Step 2					3.51 (3,34)	.026	.24	4.91 (1,34)	.11	**.033**
Age	−0.24	0.25	−0.16	.332						
BMI	−0.54	0.75	−0.11	.479						
Group	−14.17	6.39	−0.37	**.033**						
Step 3					5.33 (4,33)	.002	.39	8.48 (1,33)	.16	**.006**
Age	−0.26	0.22	−0.17	.263						
BMI	−0.44	0.68	−0.09	.525						
Group	−14.24	5.79	−0.37	**.019**						
Empathic concern	1.68	0.58	0.40	**.006**						
Step 4					6.91 (5,32)	<.001	.52	8.42 (1,32)	.13	**.007**
Age	−0.29	0.20	−0.19	.158						
BMI	−0.29	0.62	−0.06	.639						
Group	47.36	21.86	1.23	**.038**						
Empathic concern	4.10	0.98	0.97	**<.001**						
Group × empathic concern	−3.37	1.16	−1.73	**.007**						

*Note.* RMSSD = Root mean square of successive heart interbeat differences; BMI = Body Mass Index; Group = Controls or Offenders.

Bold values are significant at *p* < .05.

### Exploratory Analyses of Incongruent Empathy Patterns among Offenders

From our results above, we can distinguish between two groups of offenders: one exhibiting *congruent (coherent) empathy patterns*, that is, in which offenders show low RMSSD and low scores on the IRI, and one exhibiting “incongruent empathy patterns,” that is, in which offenders show low level on the RMSSD but high IRI scores. The next section of our results examined in more detail the rationale for identifying these groups and what could differentiate them.

We observed strong relationships between RMSSD with both self-reported Perspective Taking and Empathic Concern in the control group. These relationships were absent in the offender group (see Table 4). When examining Figure 1, we observed that the overwhelming majority of offenders (*N* = 14, 78%) had below average RMSSD, but with considerable heterogeneity in self-reported empathy. Focusing on offenders with below average RMSSD, in Figure 1a, we see that 39% (*N* = 7) of the offenders exhibit congruent responding (i.e., below average RMSSD and below average Perspective Taking scores), and 39% (*N* = 7) exhibit incongruent responding (i.e., below average RMSSD and above average Perspective Taking scores). Similarly, in Figure 1b, 33% (*N* = 6) of the offenders exhibit congruent responding (i.e., below average RMSSD and below average Empathic Concern scores), and 44% exhibit incongruent responding (i.e., below average RMSSD and above average Empathic Concern scores). To gain insight as to what might distinguish between the congruent and incongruent offender subgroups, we compared the two subgroups across all demographic and clinical variables (see Tables 1 and 2), using Mann-Whitney *U* Tests. With respect to Perspective Taking (Figure 1a), we found that incongruent offenders committed significantly more offences than congruent offenders (*Z*_(U=4.5)_ = 2.33, *p* = .020, *d* = 1.60). With respect to Empathic Concern (Figure 1b), we found no differences between the congruent and incongruent offenders.

## Discussion

The study aimed to assess the practical significance of HRV as a potential physiological correlate of empathy in comparison to a traditional self-report questionnaire of empathy in incarcerated violent offenders. There were three main findings: (1) absence of differences between offenders and non-offenders in self-reported empathy; (2) concordance between self-reported empathy and HRV among general population males; and (3) incongruent self-reported empathy and HRV in violent male offenders. We discuss each of the findings in turn.

### Absence of Differences between Offenders and Non-Offenders in Self-Reported Empathy

We found no significant differences between the offender and control groups in any of the subscale scores of the IRI. This finding is all the more surprising given evidence suggesting that protracted period of incarceration—in our sample, 7 years on average, have elapsed between the commission of the offence and testing—may result in the erosion of empathy (see Cale, 2006). Nonetheless, this result is consistent with previous reports, which failed to discern differences between offenders and non-offender groups using self-reported empathy measures, including the IRI (Domes et al., 2013; Mayer et al., 2018). This finding thus may constitute further evidence regarding the limited utility of self-reported measures alone, especially in clinical or legal settings (Hanel & Vione, 2016).

However, other potential explanations should be considered. For example, it is possible that the offenders may be just as empathetic as the controls, at least with respect to a self-reported questionnaire such as the IRI. Unfortunately, we are unable to distinguish whether these normative IRI scores among the offenders would also have been obtained at the time of the commission of the crime, or whether they are the consequence of therapeutic follow-up during detention, for example. Nonetheless, the at rest PNS levels, that is, the physiological resources that usually promote the understanding of the other’s mental states (Lischke et al., 2018; Quintana et al., 2012), are quite low for the majority (78%) of the offenders in our sample. Thus, it is also possible to speculate that these normative empathic scores in this violent population are either representative of the understanding of the mental states made possible by a compensatory biological resonance mechanism, or that these normative empathic scores are simply a reflection of *knowing (learning)* (Robinson & Rogers, 2015) without actually *being* empathic (Day et al., 2010).

### Concordance between Self-Reported Empathy and HRV in General Population Males

Consistent with our hypothesis, self-reported empathy, and specifically Perspective Taking and Empathic Concern scores were directly related to individual variations in parasympathetic nervous system activity at rest, measured with RMSSD. However, this relationship was evident in the control group only. This result is consistent with the Polyvagal Theory, which posits a close relationship between parasympathetic activity, social engagement and prosocial behaviors (Porges, 1997, 1998, 2001), with the Davis’ model (Davis, 2018), which argues that empathy could be grounded in fundamental biological constants at rest, as well as with reports showing that individual baseline parasympathetic activity can predict variations in social ability (Barry & Sokolov, 1993; Lischke et al., 2017; Mathersul et al., 2013).

### Incongruent Self-Reported Empathy and HRV in Violent Male Offenders

The lack of association between HRV and self-reported empathy in offenders was characterized with a marked PNS withdrawal in the majority of offenders, and with a considerable heterogeneity in self-reported empathy. This led us to suspect that the offender group may be composed of at least two subgroups: those with low RMSSD and low IRI, that is, the congruent group, and those with low RMSSD and high IRI, that is, the incongruent group (see Figure 1). Our exploratory analyses comparing the two groups showed that offenders with low RMSSD and high Perspective Taking scores were arrested for crimes involving a higher number of infractions. While tentative, this finding is interesting because intact cognitive empathy in these offenders is reminiscent of offenders with elevated psychopathic traits (Abu-Akel & Abushua’leh, 2004; Abu-Akel et al., 2015; Gillespie et al., 2020; Shamay-Tsoory et al., 2010), who are known for their abilities to manipulate their victims. However, multiple infractions in the same course of action (e.g., driving without a license, plus illegal carrying of weapons, substance abuse and physical assault) could be typical of impulsive and antisocial profiles, without engaging empathy or concern for the well being of others (Bjørkly, 2013; Ramírez & Andreu, 2006). By extension, it can be argued that the preservation of cognitive empathy in violent offenders may contribute to recidivism and the persistence of criminal behavior (Martin et al., 2019).

### Parasympathetic Activity: A Biomarker of Social and Mental Health

Our results show that whereas self-reported empathy failed to discriminate incarcerated violent offenders from healthy controls, they were distinguishable on measures of HRV. This is consistent with previous research showing that a massive weakness in the PNS inhibition system would allow for the dominance of the SNS “alarm” system, which in the long term could be deleterious to overall mental and somatic health (Brook & Julius, 2000; Thayer & Friedman, 2004). This profile is evident in our offender sample, whose parasympathetic activity withdrawal was accompanied with high sympathetic activity (see Table 3). However, given that 68% of our sample is psychopathological (see Table 1), it could also be argued that low parasympathetic activity could also be more generally a biomarker of a range of psychopathologic disorders (Thayer & Brosschot, 2005). Thus, to fully assess the role and specificity of the autonomic nervous system in violent offending, future research should implement a two (low/high parasympathetic activity) × two (low/high sympathetic activity) × two (presence/absence of psychopathology) design.

### Limitations and Future Directions

The results of this study should be considered in the light of its limitations. First, offenders were tested while in jail, whereas controls were not. It is possible that differences in the living conditions may be a source of variance in the HRV of the two groups. However, even though detention is by definition restrictive, it has been shown that long-term inmates (about 7 years on average in our study) tend to develop coping strategies and exhibit lower levels of stress than new prisoners (MacKenzie & Goodstein, 1985), or compared to offenders in pre-trial detention awaiting judgment (Taylor et al., 2010). Accordingly, it is possible that the potential effect of the environment (setting) on HRV in our study may have been mitigated. Second, our sample is relatively small, and thus the robustness of our findings should be ascertained in a larger sample. Third, while the offender and control groups are rather well matched, future research should consider the inclusion of additional factors, such as IQ and cognitive skills, that may moderate the relationship between HRV and empathic responses (Thayer et al., 2009). Fourth, since our sample consisted of male-only participants, our findings may be not be generalizable to females in light of evidence for sex-dependent relationship of resting state HRV with emotion regulation and empathic concern (Lischke et al., 2019; Tracy & Giummarra, 2017). Fifth and finally, given evidence for age-dependent changes in empathic responding (O’Brien et al., 2013) and HRV (Yukishita et al., 2010), age-controlled longitudinal studies would be interesting in order to assess the co-evolution of empathy and HRV in prison in comparison to the general population across the life span.

## Conclusion

We identified a robust relationship between parasympathetic activity, indexed with the RMSSD of HRV, and empathy among healthy males. This relationship was absent in severe male violent offenders. Establishing the validity of HRV as a measure of empathy should be a research priority, particularly if a sizable minority of offenders is adept at masking or camouflaging empathic deficits (Robinson & Rogers, 2015). Indeed, in our sample, we observed that while the majority of offenders presented weak parasympathetic activity, almost half of them showed above average self-reported empathy levels. It would be important for future investigations to determine whether these skills in this group are a sign of psychopathy and therefore increased risk of violent offending, or whether these skills can be leveraged to protect against violence and recidivism. Finally, as evident from their low empathy scores and low HRV, a third of the prisoners may be deprived of the biological resources and skills necessary to understand and “deploy” empathy. Further studies involving a larger number of individuals are needed to investigate this particular group. To make further progress in understanding the interrelationship between the autonomic nervous system, empathy and violent offending, it will be necessary to evaluate these aspects in individuals with various psychopathological and autonomic nervous system profiles. The present study highlights the utility and potential of such an approach.
